# The first proven oxychilid land snail endemic to China (Eupulmonata, Gastrodontoidea)

**DOI:** 10.3897/zookeys.870.32903

**Published:** 2019-08-06

**Authors:** Min Wu, Zhengping Liu

**Affiliations:** 1 School of Life Sciences, Nanjing University, Nanjing 210023, China Nanjing University Nanjing China; 2 Huarun 24 Town, Chenghuaqu, Chengdu 610051, China Unaffiliated Chengdu China

**Keywords:** Geometric morphometric analysis, ITS2, new genus, Oxychilidae, phylogeny, Sichuan

## Abstract

A new and the first proven oxychilid species endemic to China is reported from Sichuan Province. *Sinoxychilus***gen. nov.** is established based on this new species and has diagnostic traits of the sculptured protoconch, partial epiphallus wrapped by developed penis sheath, penial retractor muscle inserting on the top of penial caecum, spinelets on penial pilasters, absence of epiphallic papilla and perivaginal gland present on vagina and proximal bursa copulatrix duct. In light of shell morphology and through geometric morphometric analyses, *Zonites scrobiculatus scrobiculatus* Gredler and *Z. scrobiculatus hupeina* Gredler are proposed to be included in the new genus. A phylogenetic inference based on ITS2 gene indicates that the new genus is systematically close to *Oxychilus* Fitzinger, which is known from the Western Palearctic and the Southwestern Arabian Peninsula, regions that are geographically far from the distribution range of the new genus.

## Abstract (cn)

首次从四川青城山报道了璃螺科（Oxychilidae，癞蜗牛总科Gastrodontoidea）的陆生软体动物。依据胚螺具雕饰、交接器鞘包裹部分成荚器、交接器收缩肌着生于交接器盲囊顶端、交接器内的壁柱具小棘、成荚器乳突阙如、雌道及纳精囊管基部具雌道周腺等特征，建立了由貊华璃螺*Sinoxychilus melanoleucus***gen. nov. and sp. nov.**为属模式种的华璃螺属*Sinoxychilus***gen. nov.**。根据贝壳特征及贝壳几何形态学分析，认为分别分布于湖南和湖北的*Zonites scrobiculatus scrobiculatus* Gredler, 1885 与*Z. scrobiculatus hupeina* Gredler, 1887当归于本新属。据ITS2序列重建的支序关系揭示该属与广泛分布于古北区西部等地区的璃螺属（*Oxychilus* Fitzinger）具有较其它癞蜗牛总科类群更近的系统发生关系。地理分布上，华璃螺为位于璃螺科连续分布区之外的一属。

## Introduction

The family Oxychilidae Hesse, 1927 is one of the three families under the superfamily Gastrodontoidea Tryon, 1866 (sensu Bouchet et al. 2017) and is distributed in the Western Palearctic and the Southwestern Arabian Peninsula (Neubert 1998; Schileyko 2003). The members of this family in China were believed to belong to the genus *Hyalina* A. Férussac, 1821 (= *Oxychilus* Fitzinger, 1833). Möllendorff reported a *Hyalina* sp. from the mountains at Kiukiang (= Jiujiang, Jiangxi) (1875a) and *H. fulva* Müller, 1774 from Po-chwa-schan (= Baihuashan, Beijing) (1875b).

Gredler (1881) proposed H. (Conulus) franciscana Gredler, 1881 and its subspecies H. (Conulus) franciscana planula Gredler, 1881 (Futschiazung, Hunan) (Gredler 1881a), and treated *Helix rejecta* L. Pfeiffer, 1859 (N and Central China, Guangdong) as a *Hyalina* species (Gredler 1881a, 1881b). Later, Gredler (1882) proposed two additional species, Hyalina (Conulus) spiriplana Gredler, 1882 (Fu-tschiao-zung, Hunan) and *H.* (*Zonitoides*?) *loana* Gredler, 1882 (Changsha, Hunan) (Gredler 1882a) and listed more than 10 species, namely *H. politissima* (L. Pfeiffer, 1853) (NE China, but originally described from Sri Lanka), *H. superlita* (Morelet, 1862) (Whampoa and Canton), *H. rejecta* (NE China, Hunan, Guangdong), *H. moellendorffi* (Reinhardt, 1877) (Peking), *H. perdita* (Deshayes, 1874) (Near Peking, Eastern Mongolia), H. (Conulus) franciscana, H. (Conulus) franciscana planula (Hunan), and H. (Conulus) fulva (Peking and the Eastern Mongolia) (Gredler 1882a, 1882b). Heude (1882) described 13 new species of *Hyalina*, namely *H. planula* (Ningguo, Anhui), *H. rathouisii* (near Shanghai), *H. planata* (Hunan), *H. mamillaris* (Hunan), *H. sinensis*, *H. zikaveiensis* (Shanghai), *H. sekingeriana* (Ningguo, Anhui), *H. colombeliana* (Yixing, Jiangsu), *H. bambusicola* (Ningguo, Anhui), *H. spelaea* (Guanyinmen, Nanjing, Jiangsu), *H. castaneola* (Qingyang, Anhui), *H. imbellis* (Ningguo, Anhui), and *H. gredleriana* (Hunan), and treated *Helix rejecta* and *Helix miliaria* Gredler, 1881 as species of *Hyalina*. Retaining *H. politissima*, *H. planula*, and *H. zikaveiensis* (“*Likaveiensis*” was a typographical error by Möllendorff 1883: 375) in *Hyalina*, Möllendorff (1883) arranged part of above mentioned species, most proposed by Heude (1882), into four genera, viz. *H. superlita* in *Macrochlamys* Benson, 1832; *H. rejecta* (synonym *H. mamilaris*), *H. planata*, *H. moellendorffi*, *H. sinensis*, *H. colombeliana*, *H. sekingeriana*, *H. bambusicola*, *H. spelaea* and *H. castaneola* in *Microcystis*? Beck, 1837; *H. imbellis*, *H. franciscana* and *H. gredleriana* in *Kaliella*? Blanford, 1863; and *H. rathouisii* in *Nanina* Grey, 1834.

Gredler (1885) proposed *H. crystallodes* Gredler, 1885. In the second series of the “Manual of Conconchology”, Tryon (1886) included *H. gredleriana* as a subspecies of *H. franciscana*. He categorized the *Hyalina* species into seven genera (= sections), placing *H. franciscana*, *H. franciscana gredleriana*, and *H. imbellis* under *Kaliella*; placing *H. politissima*, *H. sinensis*, *H. superlita* Morelet, 1862 (Hongkong, Macao, Kuang-tung), and *H. rathouisii* in *Macrochlamys*, moving *H. zikaveiensis*, *H. planula*, *H. sekingeriana*, *H. colombeliana*, *H. spelaea*, *H. castaneola*, *H. bambusicola*, *H. ejecta*, *H. planata*, and *H. moellendorffi* to *Microcystis*; moving *H. mamillaris* and *H. perdita* to *Polita* Held, 1837, placing *H. loana* in *Zonitoides* Lehmann, 1864; and moving *H. spiriplana* and *H. fulva* to *Conulus* Fitzinger, 1833.

Then, Gredler transferred *H. franciscana*, *H. franciscana planula* Gredler (not Heude, 1882), and *H. spiriplana* to *Kaliella* and transfered *H. rathouisii* to *Nanina*; while retaining *H. politissima*, *H. planula* Heude, *H. zikaveiensis*, and *H. loana* in *Hyalina* (Gredler 1887a). Yen (1939) grouped *H. spelaea*, *H. sekingeriana*, and *H. franciscana* into *Kaliella* Blanford, 1863 and *H. zikaveiensis* (in Yen 1939: 118, “*sicaveiensis*” was a typographical error) into *Microcystina* Mörch, 1876; he placed *H. sinensis*, *H. planula*, *H. planata*, and *H. rejecta* in *Macrochlamys* and moved *H. rathouisii* to *Euplecta* Semper, 1870.

None of the above mentioned species was anatomically examined. Over-dependence on shell morphology caused many conflicts in the early classification of Chinese species of *Hyalina*. Furthermore, none of the above-mentioned species that had once been treated as *Hyalina* has been studied since Yen (1939), and the existence of true oxychilid species in China has been questioned. However, our recent work on the malacofauna of Sichuan, has found a species which meets the morphological definition of Oxychilidae Hess, 1927 but conchologically differs from above-mentioned *Hyalina* species. The close relationship of the new genus with the oxychilid *Oxychilus* is also supported by molecular data.

## Materials and methods

Four living animals and three empty shells, all fully mature, were collected by hand from the type locality. The living specimens were relaxed by drowning in water before being transferred to 70% ethanol which was replaced with ethanol of the same concentration after three days. The sizes of shell and genitalia of each specimen were measured with calibrated digital Vernier callipers and from photos, both to the nearest 0.1 mm. The number of whorls was recorded with 0.125 whorl accuracy as described by Kerney and Cameron (1979). Soft parts were measured after the specimens were fixed in 70% ethanol.

Whole genomic DNA was extracted from a piece of pedal muscle of the ethanol-preserved specimens using Animal Genome Quick Extraction Kit (B518221, Sangon Biotech). Each 25 μL PCR mixture consisted of 12.5 μL cwbio 2× Es Taq MasterMix Dye, 9.5 μL ddH_2_O, 1 μL template DNA, 1 μL forward primer (10μL/L) (5′-CTAGCTGCGAGAATTAATGTGA-3′, Wade and Mordan 2000) and 1 μL reverse primer (10 μL/L) (5′-ACTTTCCCTCACGGTACTTG-3′; Wade and Mordan 2000). The conditions for thermal cycling, performed on a Eastwin ETC811, was 2 min at 94 °C for pre-denaturing, 35 circles of 30 s at 94 °C, 30 s at 58 °C and 60 s at 72 °C. The amplicons were examined on a 1% agarose gel for quality and fragment size, then were purified and sequenced on an automated sequencer. Information of the outgroup in phylogenetic inference: *Pseudiberus liuae* Wu, 2017 (Camaenidae), 33.102N, 104.336E, Shijiba, Wenxian, Gansu Province, China; June 10, 2011; coll. Wu, M., Xu, Q. & Buhda, P., registered and DNA voucher no. HBUMM06758.

Chromatographs and sequences were examined and were initially compiled in Sequencher 4.5. The sequence alignment, the evolution model selection and the Maximum Likelihood inference were performed by MEGA 7.0.26 (Kumar et al. 2016). After the data set of internal transcribed spacer 2 (ITS2) were examined by Gblocks 0.91b (Castresana 2000), 58% of the original 950 positions was retained for the final phylogenetic analyses. The Bayesian inference was conducted using MrBayes 3.1 (Ronquist et al. 2012).

Shell morphological variation study was performed in the tps series software including tpsUtil32 (Rohlf 2004), tpsDig32 (Rohlf 2005), using the geometric morphometric (GM) methods based on the landmarks (LMs) and semi-landmarks on the contour of the shell in aperture view (Schilthuizen et al. 2012). The designs of the landmarks and semi-landmarks are as follows: LM1, the columella insertion; LM2, the right insertion of peristome onto body whorl; LM3, the intersection point of right contour and suture of the last whorl; LM4 and LM8, respective right and left extremities on suture; LM5 and LM7, the right and left extremities on suture above LM4 and LM8, respectively; LM6, apex of shell; LMs 9–26, 18 semi-landmarks on the left contour between LM8 and the intersection point of left contour with peristome, by length; LMs 27–44, 18 semi-landmarks on peristome between LM1 and LM2, by length (Fig. 7; the number on landmarks transferred from semi-landmarks are not shown). The landmarks and the semi-landmarks were treated indiscriminately. The geometric morphometric analysis employed photos of 32 shells in aperture view, including five type specimens of the new species described in this paper, 10 Indian *Ariophanta* species randomly selected from Raheem et al. (2014), and 15 oxychiline species randomly selected from Sysoev and Schileyko (2009). Full Procrustes fitting, covariance matrix generating, and subsequent canonical variate analysis (CVA) were conducted using MorphoJ (version 1.05f; Klingenberg 2011).

Directions used in descriptions: proximal = towards the genital atrium; distal = away from the genital atrium.

Abbreviations: **At** – atrium; **BC** – bursa copulatrix; **BCD** – bursa copulatrix duct; **Ep** – epiphallus; **fma** – fully mature animal(s); **FO** – free oviduct; **HBUMM** – Mollusc collection of the Museum of Hebei University, Baoding, China; **OE** – orifice of epiphallus; **P** – penis; **PC** – penial caecum (this part is judged here as penial caecum rather than flagellum as termed in some works because the flagellum, if present, is located at the distal end of epiphallus); **PP** – penial pilaster; **PR** – penial retractor muscle; **PS** – penis sheath; **PVG** – perivaginal gland; **Va** – vagina; **VD** – vas deferens.

## Systematics

### Gastrodontoidea Tryon, 1866

#### Oxychilidae Hesse, 1927

##### Oxychilinae Hesse, 1927

###### Sinoxychilus gen. nov.

Taxon classificationAnimaliaStylommatophoraOxychilidae

F57CC4A72CA05514A2493F8488905119

http://zoobank.org/C90C05C0-17A9-4D50-BC87-25688D997E07

####### Type species.

*Sinoxychilus melanoleucus* gen. nov. and sp. nov.

####### Diagnosis.

Protoconch with intercrossing radial wrinkles and spiral grooves. Penis sheath developed, more or less wrapping partial epiphallus. Tubercles of broken longitudinal penial pilasters bearing spinelets. Penial retractor muscle inserting on the top of penial caecum. Neither flagellum nor epiphallic papilla present. Perivaginal gland present on vagina and proximal bursa copulatrix duct.

####### Description.

Shell depressed; thin; opaque; of about 4.5 whorls. Umbilicus moderately wide. Protoconch with intercrossing radial wrinkles and spiral grooves. Teleoconch with spiral furrows. Aperture somewhat sinuate at peristome. Aperture toothless, unexpanded.

Sole tripartite. Caudal foss or caudal horn absent. Jaw oxygnathous, with median projection.

Penis sheath present; wrapping partial epiphallus. Penis moderately long and thick; externally simple. Sarcobelum absent. Penial caecum present, having no external demarcation between it and penis. Penial retractor muscle inserting on top of penial caecum. Flagellum absent. Epiphallus thin. Penial caecum internally with transversal ridges near epiphallic pore. Epiphallic papilla absent. Penis internally with developed pilasters. Penial pilasters broken into connected tubercles that each bearing a very short spinelet. Vagina short, internally simple, and without papilla or verge. Perivaginal gland well developed on the surface of vagina and proximal part of bursa copulatrix duct.

####### Distribution.

China (Sichuan, Hunan, Hubei).

####### Etymology.

The generic name is a compound of Greek “sino” (= China) and *Oxychilus* which is a genus of the family Oxychilidae.

####### Molecular phylogenetic analyses.

The examined ITS2 sequences are from GenBank and this study. According to Hausdorf (2000), Gastrodontoidea is made up of six families, namely Pristilomatidae Cockerell, 1891, Chronidae Thiele, 1931, EuconulidaeH.B. Baker, 1928, Trochomorphidae Möllendorff, 1890, Gastrodontidae Tryon, 1866, and Oxychilidae. After searching for ITS2 sequences from these six families in NCBI (https://www.ncbi.nlm.nih.gov/), 21 ITS2 haplotypes of *Euconulus* spp. (Euconulidae), *Oxychilus* spp. (Oxychilidae), and one *Vitrea* species (Pristilomatidae) were added to our analyses (Table 1). After eliminating poorly aligned positions and divergent regions of the alignment, a dataset of 25 × 552 bp was used for the subsequent analyses. The “T92 (Tamura 3-parameter) + G” model was chosen as the best nucleotide substitution model because of the lowest AIC score (lnL = −1746.871, AICc = 3594.112). The phylograms produced by the Maximum Likelihood Inference and the Bayesian Inference are topologically identical (Fig. 8). The obtained phylogenetic inference shows *Sinoxychilus* gen. nov. forms a sister group with the genus *Oxychilus*, and both genera are well embedded in the Gastrodontoidea clade (Fig. 8).

**Table 1.** The species and ITS2 sequences used for phylogenetic study.

**Family**	**Species**	**Genbank Accession No. of ITS2**
Euconulidae	*Euconulus alderi* (J.E. Gray, 1840)	MK299689, MK299710
	*E. chersinus* (Say, 1821)	MK299741
	*E. dentatus* (Sterki, 1893)	MK299732, MK299739
	*E. fulvus* (O.F. Müller, 1774)	MK299691, MK299693, MK299695, MK299702, MK299723, MK299724, MK299737, MK299738
	*E. trochulus* (Reinhardt, 1883)	MK299730–31
	*E. polygyratus* (Pilsbry, 1899)	MK299747
Pristilomatidae	*Vitrea crystalline* (O.F. Müller, 1774)	AY014113
Oxychilidae	*Oxychilus alliarius* (Miller, 1822)	JF837183, AY014114
	*O. cellarius* (O.F. Müller, 1774)	AY014116
	*O. helveticus* (Blum, 1881)	AY014115
	*Sinoxychilus melanoleucus* gen. nov. & sp. nov.	MN056416, MN056417
Camaenidae (Outgroup)	*Pseudiberus liuae* Wu, 2017	MN056414, MN056415

####### Taxonomic remarks.

Morphologically, this group belongs to the family Oxychilidae based on the presence of a tripartite sole, oxygnathous jaw, penis sheath, and perivaginal gland and the absence of a caudal horn and sarcobelum, by which *Sinoxychilus* gen. nov. can be promptly distinguished from Gastrodontidae and Pristilomatidae, the other two families of Gastrodontoidea (sensu Bouchet et al. 2017). The new genus and *Oxychilus* have many characteristics in common, such as a developed penial caecum, connection of some part of epiphallus + vas deferens and distal penis sheath by connective tissue, as in the European *Oxychilus mortilleti* (L. Pfeiffer, 1859) (Manganelli and Giusti 1998: figs 5, 10, 13, 14) and in the Asian *Araboxychilus sabaeus* (Martens, 1889) (Colville and Riedel 1998: fig. 7). However, *Sinoxychilus* gen. nov. differs from *Oxychilus* in having an opaque shell with a delicately sculptured protoconch, and in bearing short spinelets on the penial pilasters. The new genus also shows an unusual shell shape, which differs from shells of *Ariophanta* Desmoulins, 1829 and some other oxychiline genera (Fig. 7).

*Zonites scrobiculatus scrobiculatus* Gredler, 1885 and *Z. scrobiculatus hupeina* Gredler, 1887 are included in the new genus although they are only known conchologically (see Taxonomic remarks below).

*Riedeliconcha* Schileyko, 2003 and *Vitrinoxychilus* Riedel, 1963 are two oxychilid genera which also have spines on the penis inner wall. The new genus differs from them in possessing well-developed penial caecum, penis sheath, and epiphallus, a long bursa copulatrix, and conchologically, an opaque shells with a sculptured protoconch.

###### Sinoxychilus melanoleucus

Taxon classificationAnimaliaStylommatophoraOxychilidae

gen. nov. & sp. nov.

93DE6CE39077552587BB0FA5DDAB0AE4

http://zoobank.org/E4075613-A987-471A-A095-D058CBDA466F

Figures 1 , 2 , 3 , 4 , 5 , 6 , 7 , 8 , 9 , 10 , Table 1

####### Type material.

**Holotype**, 1 fma (HBUMM08236 specimen-1), Qingchengshan Mt., humid forest, in litter (Fig. 9); Sichuan Province, China; 30.919N, 103.494E; 24 March 2018; coll. Liu, Zhengping & Ma, Hongwen. **Paratypes**, 3 fma (HBUMM08236; specimens 2–4) and 2 fully mature empty shells (HBUMM08236; specimens 6, 7), collection data as holotype. One empty shell specimen (HBUMM08236; specimen 5) was broken accidentally after measurement and as a result is not included as a paratype. From each of type specimens with soft parts (HBUMM08236; specimens 1–4) a piece of foot (HBUMM08236a; specimens 1–4) was cut and preserved in 99.7% ethanol at −20 °C.

####### Description.

Shell (Figs 2, 3). Dextral; clearly depressed; very thin and fragile; opaque. Whorls convex. Suture impressed. Umbilicus moderately wide. Basal-umbilicus transition gentle. Columella arched to oblique. Columellar lip not dilated, never covering umbilicus. Protoconch with intercrossing radial wrinkles and spiral grooves (Fig. 3A). Teleoconch with regularly, densely distributed spiral furrows (Fig. 3B). Growth lines fine, distinct. Aperture large, oblique, somewhat sinuate at peristome. Body whorl straight. Adult shell neither hairy nor scaly. Body whorl of adult shell very bluntly angulate at periphery, with base convex. Aperture toothless, unexpanded. Peristome rather thin. Callus indistinct. Shell in uniformly greenish yellow, spiral band absent (Fig. 2). Measurements (*n* = 6): shell height = 6.7–8.1 (7.7 ± 0.55) mm, shell breadth = 12.6–13.8 (13.2 ± 0.51) mm, aperture height = 4.9–5.7 (5.3 ± 0.31) mm, aperture width = 2.2–2.6 (2.4 ± 0.16) mm, embryonic shell whorls = 1.38–1.63 (1.50 ± 0.079) mm, whorls = 4.25–4.63 (4.41 ± 0.151) mm, shell height/breadth ratio = 0.53–0.62 (0.58 ± 0.030) mm.

**Figure 1.**
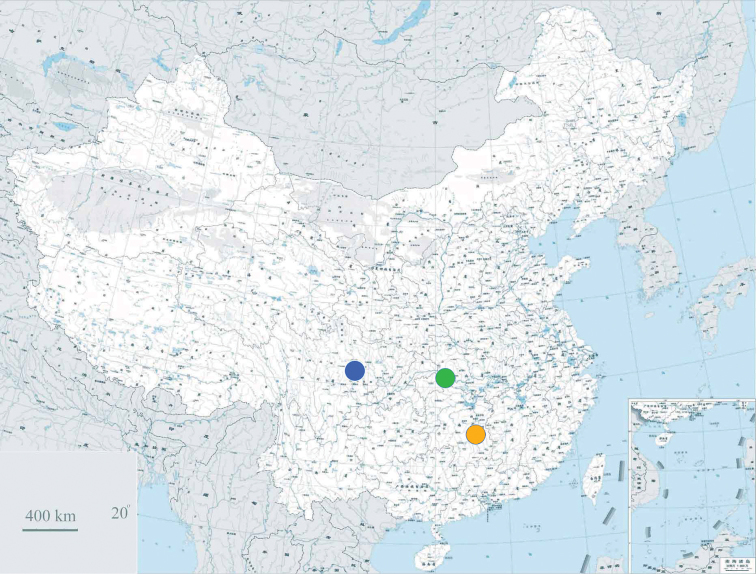
Distribution map of *Sinoxychilus melanoleucus* gen. nov and sp. nov. (blue dot), *Zonites scrobiculatus scrobiculatus* Gredler, 1885 (orange dot), and *Zonites scrobiculatus hupeina* Gredler, 1887 (green dot).

**Figure 2.**
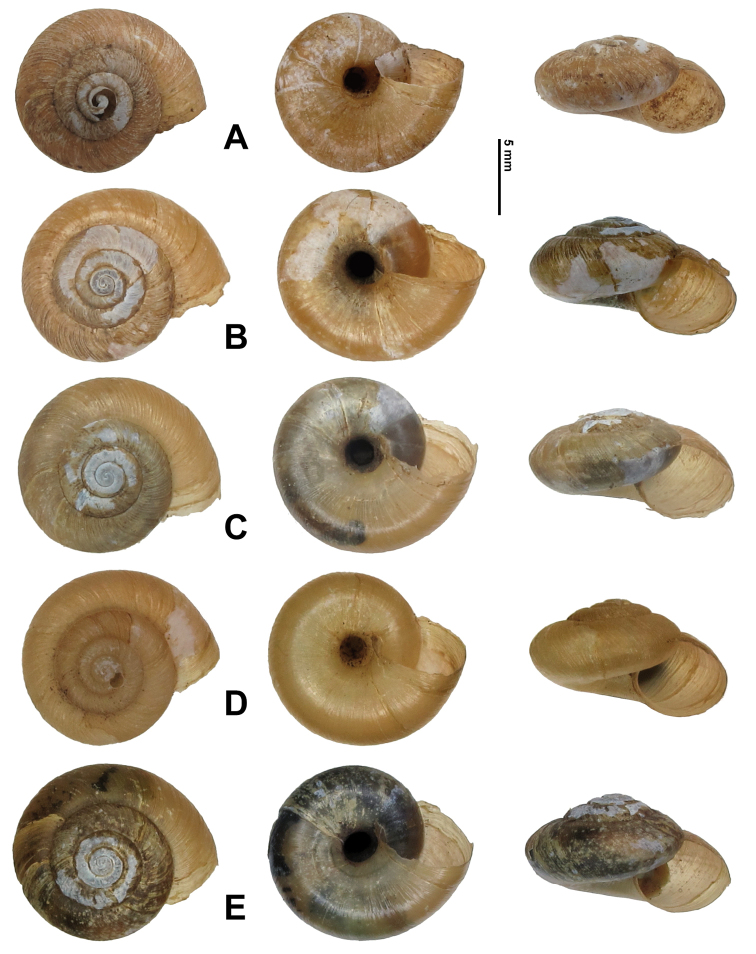
*Sinoxychilus melanoleucus* gen. nov. and sp. nov. shells. **A** Holotype, HBUMM08236 specimen 1 **B–E** paratypes, HBUMM08236 specimens 2–4, 6.

**Figure 3.**
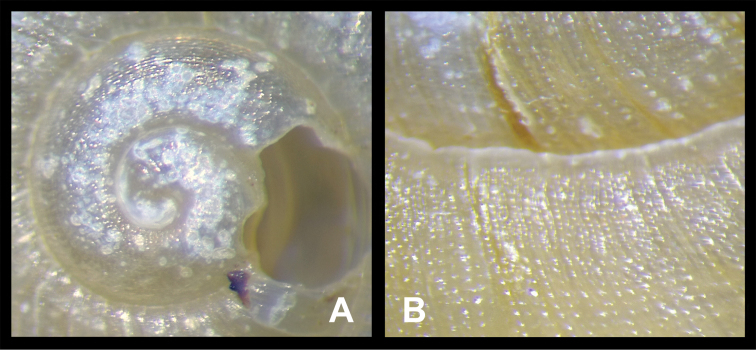
*Sinoxychilus melanoleucus* gen. nov. and sp. nov. HBUMM08236 specimen 4, paratype **A** magnified embryonic shell **B** surface of teleoconch, magnified.

**Figure 4.**
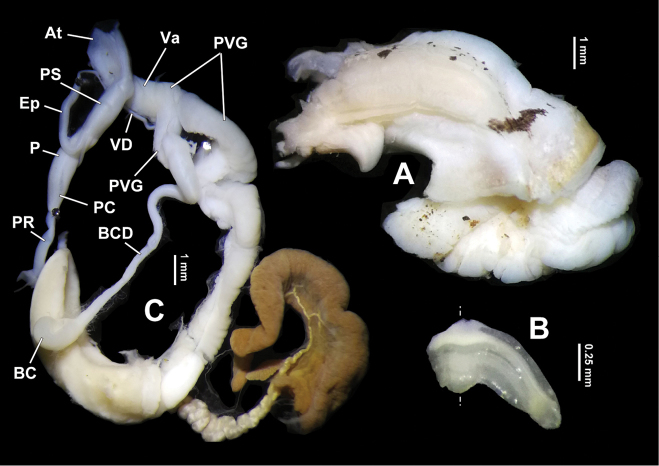
*Sinoxychilus melanoleucus* gen. nov. and sp. nov., holotype, HBUMM08236 specimen 1 **A** partial soft part **B** partial jaw. Dotted line indicating axis line **C** genitalia in general view. At-atrium; BC-bursa copulatrix; BCD-bursa copulatrix duct; Ep-epiphallus; P-penis; PC-penial caecum; PR-penial retractor muscle; PS-penis sheath; PVG-perivaginal gland; Va-vagina; VD-vas deferens.

General anatomy. Sole tripartite. Caudal foss or caudal horn absent. Eversible head wart between ommatophore insertions absent. Tentacles and dorsum leaden-black. After preservation in 70% ethanol, black pigments on animal become faint. Lower sides and sole creamy white (Figs 4A, 10). Jaw oxygnathous, with an evidently median projection (Fig. 4B).

**Figure 5.**
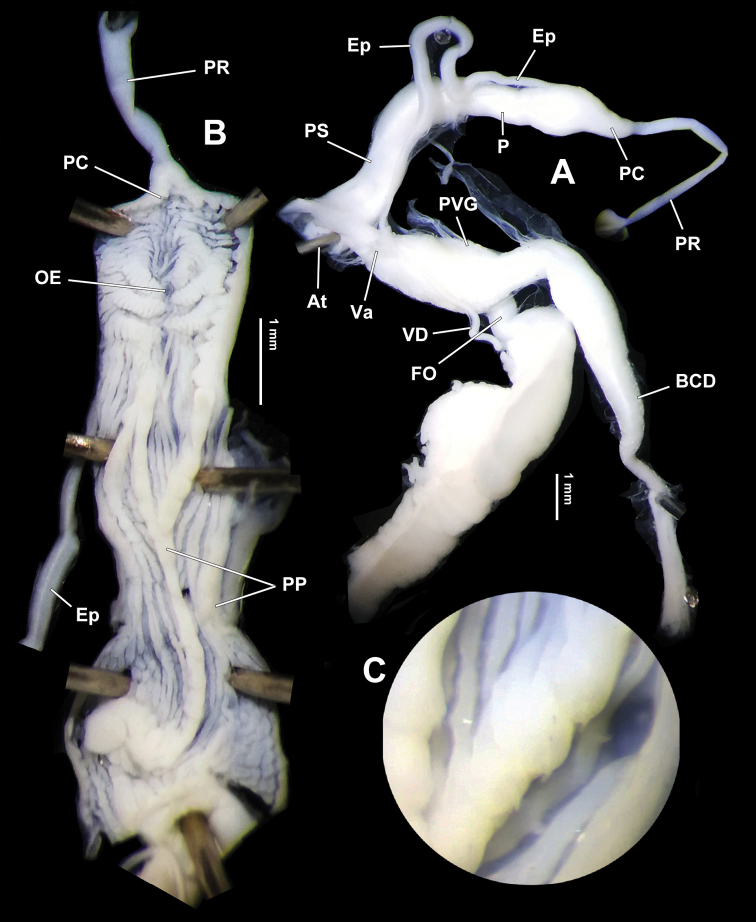
*Sinoxychilus melanoleucus* gen. nov. and sp. nov. **A** Genitalia in general view, paratype, HBUMM08236 specimen 2, showing the median section of epiphallus is not wrapped inside the penis sheath **B, C** holotype, HBUMM08236 specimen 1 **B** interior view of penis **C** a section of magnified penial pilaster, showing apical spinelet on each tubercle consisting the penial pilaster. At-atrium; BCD-bursa copulatrix duct; Ep-epiphallus; FO-free oviduct; OE-orifice of epiphallus; P-penis; PC-penial caecum; PP-penial pilaster; PR-penial retractor muscle; PS-penis sheath; PVG-perivaginal gland; Va-vagina; VD-vas deferens.

**Figure 6.**
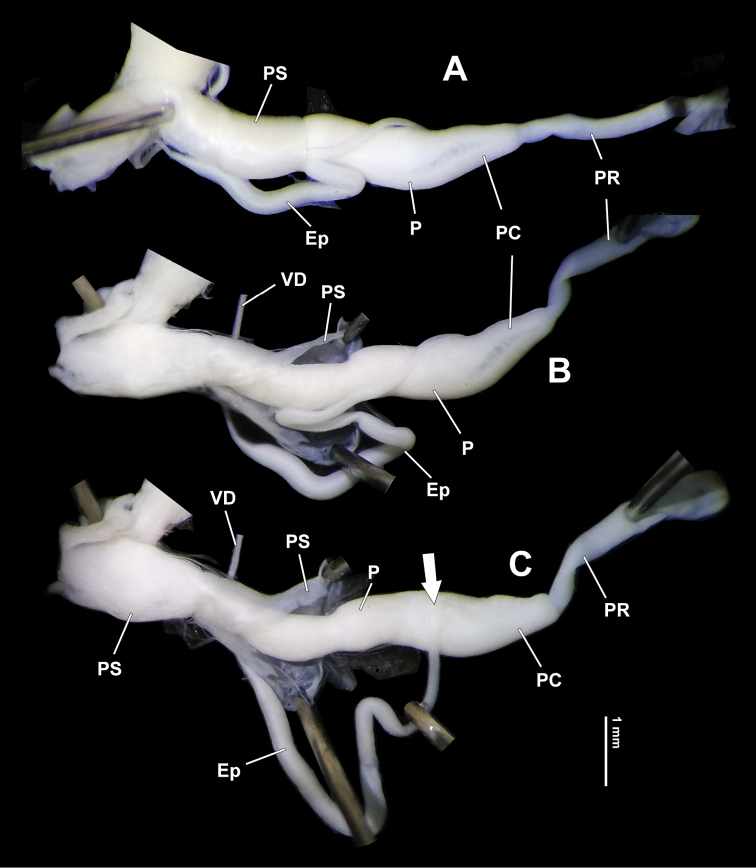
*Sinoxychilus melanoleucus* gen. nov. and sp. nov., HBUMM08236 specimen 2, paratype **A–C** indicating that the median part of epiphallus was dissecting out from the penis sheath **C** arrow indicates epiphallus insertion. Ep-epiphallus; P-penis; PC-penial caecum; PR-penial retractor muscle; PS-penis sheath; VD-vas deferens.

Genitalia (Figs 4C, 5, 6). Penis sheath about half length of penis, in holotype and two paratypes wrapping about 1/3 central epiphallus (Figs 4C, 6), but in one specimen (HBUMM08236; specimen 2) median part of epiphallus loosely joined to distal penis sheath by connective tissue (Fig. 5A). Penis more or less long, moderately thick, surface simple. Sarcobelum absent. Penial caecum present (Figs 4C, 5A, 5B, 6), having no external demarcation between it and penis (Figs 4C, 5A, 6). Penial retractor muscle inserting on top of penial caecum. Flagellum absent. Epiphallus thin, but 2–3 times thicker than vas deferens (Figs 4C, 5A, 6). Distal part of epiphallus attached at lateral side of penis by connective tissue before entering it. Penial caecum internally with three pairs of symmetrically arranged low transversal ridges near epiphallic pore which is surrounded by several very fine pilasters (Fig. 5B). Epiphallic papilla absent. Penis internally with a thickened, ‘M’-shaped median pilaster which has two arms branching into several narrow pilasters and the median pilaster running to the most proximal part of penis where it extends and forming a transversal ridge (Fig. 5B). The ‘M’-shaped median pilaster consists of connected tubercles, the apex of each bearing a very short spinelet that without exception points to atrium (Fig. 5C). Vagina short, internally simple, without papilla/verge. Perivaginal gland well developed on surface of vagina and proximal part of bursa copulatrix duct (Figs 4C, 5A). Measurements of holotype: P = 5.0 mm; Ep = 8.4 mm; VD = 6.5 mm; PR = 2.3 mm; Va = 2.3 mm; BC + BCD = 11.8 mm.

####### Distribution.

The new species is known only from its type locality.

####### Etymology.

The species is named for the clear demarcation between the leaden black ommatophores and dorsum and the remaining creamy white body, which is reminescent of the giant panda, *Ailuropoda melanoleuca* by having the color pattern of clear-cut patches of black and white (Fig. 10).

####### Ecology.

The new species was found living in extremely humid environment at type locality. In the laboratory, below 100% relative humidity, animals became active at the relatively lower temperature of 5 °C (Fig. 10) before they were totally inactive at room temperature (ca. 25 °C).

####### Taxonomic remarks.

**Figure 7.**
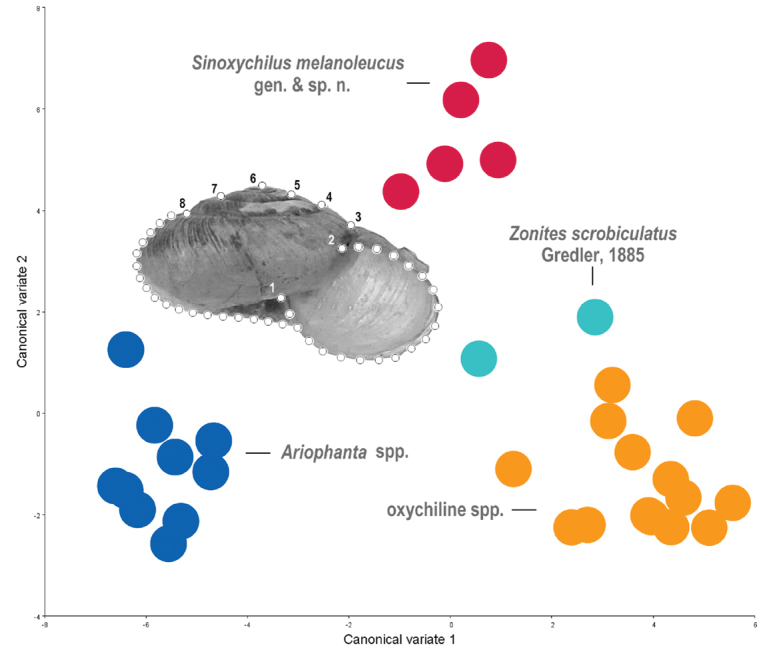
Scatter plot of canonical variate 1 against canonical variate 2 (yielded by canonical variate analysis), showing morphological relationship among *Sinoxychilus melanoleucus* gen. nov. and sp. nov. (red dots), *Zonites scrobiculatus scrobiculatus* Gredler, 1885 and *Zonites scrobiculatus hupeina* Gredler, 1887 (light blue dots), Indian *Ariophanta* spp. (Raheem et al. 2014) (dark blue dots) and oxychiline spp. (Sysoev and Schileyko 2009) (orange dots). A diagram showing design of landmarks (numbered) and semi-landmarks (not numbered) is provided.

This new species can be distinguished from all other Chinese *Hyalina* species in the measurements of its shells (Table 2) and other features. This species, however, as kindly pointed out by Dr Barna Páll-Gergely, is obviously close to *Zonites scrobiculatus* Gredler, 1885, which was usually treated as a species in the bradybaenine genus *Coccoglypta* Pilsbry, 1895 (Páll-Gergely in press). The species can be promptly distinguished from *Z. scrobiculatus*, which has two subspecies, namely *Z. scrobiculatus scrobiculatus* [*Zonites scrobiculatus* Gredler, 1885a: 220–221, pl. 6, fig. 2; Tryon 1886: pl. 53, figs 12–14; Bachmann and Gredler 1894: 416 (radula); Retinella ? scrobiculata Kobelt 1899: 918, pl. 241, figs 10, 11; *Coccoglypta scrobiculata scrobiculata*Yen 1939: 153, pl. 15, fig. 62; *Coccoglypta scrobiculata*Zilch 1974: 211; Coccoglypta (Coccoglypta)*scrobiculata scrobiculata*Zilch 1968: 180] and *Z. scrobiculatus hupeina* Gredler, 1887 [*Zonites* (*Nanina*?) *scrobiculatus var. hupeina*Gredler 1887b: 344–345; *Coccoglypta scrobiculata hupeina*Yen 1939: 153, pl. 15, fig. 63; Zilch 1974: 199; Coccoglypta (Coccoglypta) scrobiculata hupeinaZilch 1968: 180], by having a distinctly smaller shell, with fewer whorls, and a particular shell shape which is sharply divergent from that of *Z. scrobiculatus* (Fig. 7). *Sinoxychilus melanoleucus* is also geographically distant from the geographic range of *Z. scrobiculatus* (Fig. 1). Nevertheless, we are inclined to believe that based on shell morphology *Z. scrobiculatus* should belong to *Sinoxychilus*, although anatomical and molecular evidence is unavailable.

**Table 2.** Shell measurements and distribution of Chinese species once grouped in *Hyalina* A. Férussac, 1821, with synonyms excluded.

**Species**	**Whorls**	**Diam. maj. (mm)**	**Height (mm)**	**Distribution**
*Hyalina fulva* O.F. Müller, 1774	5–6	4	3.5*	Beijing, E Mongolia
*H. politissima* (L. Pfeiffer, 1853)	4.5	24	11	Sri Lanka, NE China
*H. rejecta* (L. Pfeiffer, 1859)	6	3.5	3	Hunan, Guangdong, NE China
*H. superlita* (Morelet, 1862)	5–5.5	16.5–21	10–11.5	Guangdong, Macao
*H. perdita* (Deshayes, 1874)	4	3	1.5*	Beijing, E Mongolia
*H. moellendorffi* (Reinhardt, 1877)	5.5	10	5.5	Beijing
H. (Conulus) franciscana Gredler, 1881	6	3.5	3	Hunan
H. (Conulus) f. planula Gredler, 1881	6	3.5	2	Hunan
H. (Conulus) spiriplana Gredler, 1882	4.5–5	3	1^3^/_4_	Hunan
*H.* (*Zonitoides*?) *loana* Gredler, 1882	5	4^3^/_4_–5	2	Hunan
*H. bambusicola* Heude, 1882	5	3.5–4	3.5	Anhui
*H. castaneola* Heude, 1882	6	3–4	3	Anhui
*H. colombeliana* Heude, 1882	6	4.5–5	3.5	Jiangsu
*H. gredleriana* Heude, 1882	6	2.5–3	3.5	Hunan
*H. imbellis* Heude, 1882	5.5	3.5–3^3^/_4_	3	Anhui
*H. planula* Heude, 1882	4.5	6–7	3	Anhui
*H. planata* Heude, 1882	4	9–10	3.5	Hunan
*H. rathouisii* Heude, 1882	7	6	3.5	Shanghai
*H. sekingeriana* Heude, 1882	6	3.5–3^3^/_4_	3.5	Anhui
*H. sinensis* Heude, 1882	4	5–6	3.5	Yangtze River Valley
*H. spelaea* Heude, 1882	6	4–4.5	3	Jiangsu
*H. zikaveiensis* Heude, 1882	4	2	1	Shanghai
*H. crystallodes* Gredler, 1885	5-5.5	5	2	Hunan

* Measured from two figures in Tryon (1886: pl. 53).

With respect to the genitalia, *Sinoxychilus melanoleucus* is similar to the Japanese *Urazirochlamys doenitzii* (Reinhardt, 1877) (Helicarionidae sensu Azuma 1995 and Schileyko 2002) in having the apical insertion of penial retractor and the absence of flagellum (Schileyko 2002: fig. 1600). *Sinoxychilus* and *Urazirochlamys* Habe, 1946 also share a characteristically spirally sculptured protoconch. However, the latter genus has a caudal horn (Azuma 1995: pl. 28, fig. 339), which suggests that *Urazirochlamys* does not belong to the Oxychilidae.

With the exception of two genera distributed in the southwestern part of the Arabian Peninsula, oxychilid snails are only known from the Western Palearctic (Neubert 1998; Schileyko 2003). The new species described herein, and its congeners, are undoubtedly the easternmost representatives of Oxychilidae, which suggests that *Sinoxychilus* might be an isolated group in China, remote from the main distribution area of the family.

**Figure 8.**
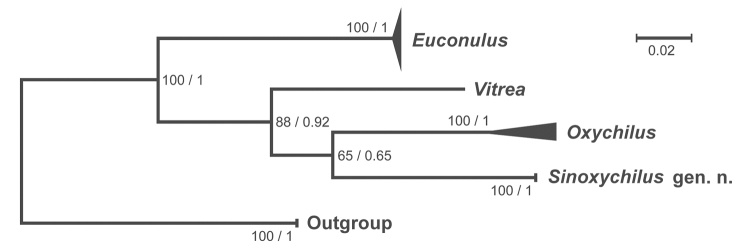
Maximum likelihood tree based on ITS2 gene (-ln likelihood = −1746.99). Ingroups: *Euconulus* Reinhardt, 1883 (Euconulidae), *Vitrea* Fitzinger, 1833 (Pristilomatidae), *Oxychilus* Fitzinger, 1833 (Oxychilidae), and *Sinoxychilus* gen. nov. (Oxychilidae). This ML tree shares the same topology with the Bayesian Inference tree. Numbers on branches indicate maximum likelihood and Bayesian posterior probabilities.

**Figure 9.**
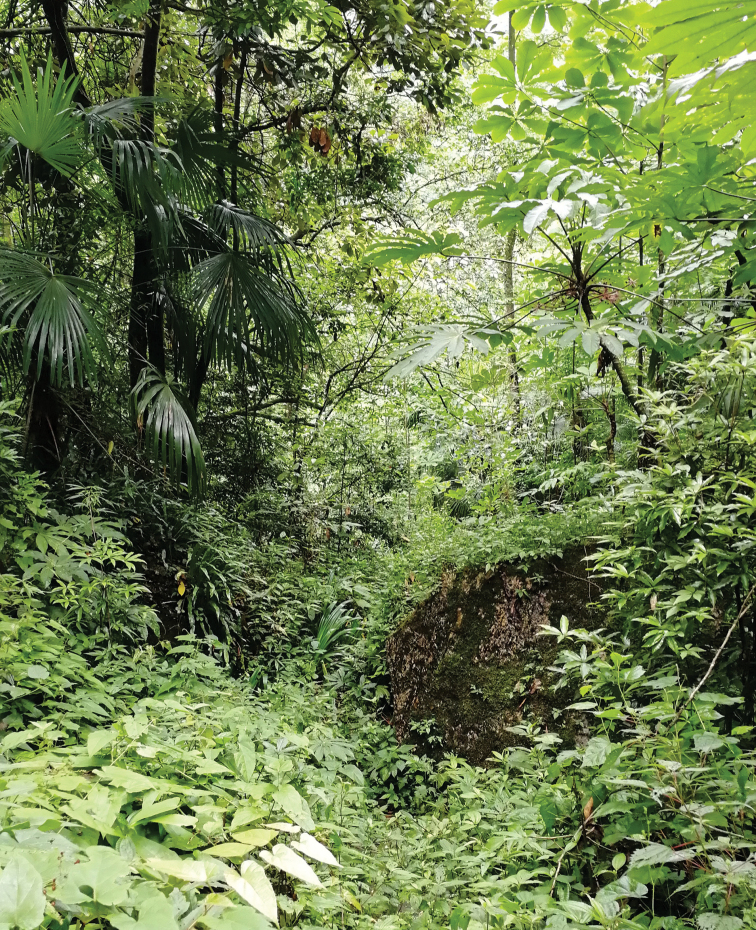
Habitat of *Sinoxychilus melanoleucus* gen. nov. and sp. nov. Qingchengshan, Sichuan.

**Figure 10.**
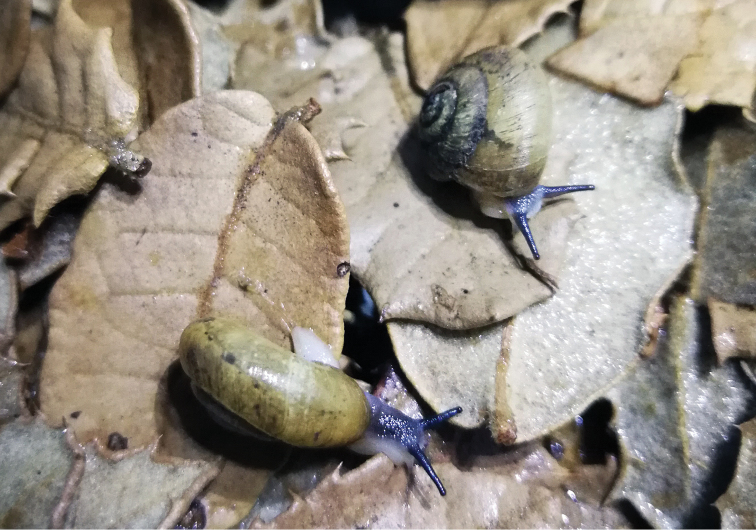
*Sinoxychilus melanoleucus* gen. nov. and sp. nov. Active animals. The photo was taken in laboratory rather than from the original habitat.

## Supplementary Material

XML Treatment for Sinoxychilus

XML Treatment for Sinoxychilus melanoleucus

## References

[B1] Azuma M (1995) Colored Illustrations of the Land Snails of Japan. Hoikusha, Osaka, 343 pp. [80 pls]

[B2] Bachmann O Gredler V (1894) Zur Conchylien-Fauna von China. XVIII. Stück. Annalen des K. K. Naturhistorischen Hofmuseums 9: 415–429.

[B3] Bouchet P Rocroi J-P Hausdorf B Kaim A Kano Y Nützel A Parkhaev P Schrödl M Strong EE (2017) Revised classification, nomenclator and typification of gastropod and monoplacophoran families. Malacologia 61(1–2): 1–526. 10.4002/040.061.0201

[B4] Castresana J (2000) Selection of conserved blocks from multiple alignments for their use in phylogenetic analysis. Molecular Biology and Evolution 17: 540–552. 10.1093/oxfordjournals.molbev.a026334 10742046

[B5] Colville B Riedel A (1998) On the systematic position of *Araboxychilus sabaeus* (Gastropoda: Pulmonata) from the South-west of the Arabian Peninsula. Journal of Conchology 36(3): 27–34.

[B6] Gredler V (1881a) Zur Conchylienfauna von China. II. Stück. Jahrbücher der Deutschen Malakozoologischen Gesellschaft 8: 10–33.

[B7] Gredler V (1881b) Zur Conchylienfauna von China. III. Stück. Jahrbücher der Deutschen Malakozoologischen Gesellschaft 8: 110–132.

[B8] Gredler V (1882a) Zur Conchylienfauna von China. IV. Stück. Jahrbücher der Deutschen Malakozoologischen Gesellschaft 9: 38–50.

[B9] Gredler V (1882b) Übersicht der Binnenschnecken von China. Malakozoologische Blätter Neue Folge 5: 165–187.

[B10] Gredler V (1885a) Zur Conchylienfauna von China. VII. Stück. Jahrbücher der Deutschen Malakozoologischen Gesellschaft 12: 219–235.

[B11] Gredler V (1885b) Zur Conchylienfauna von China. VIII. Stück. Selbstrerlag, Bozen 3–19.

[B12] Gredler V (1887a) Zur Conchylienfauna von China. X. Stück. Malakozoologische Blätter Neue Folge 9: 121–163.

[B13] Gredler V (1887b) Zur Conchylienfauna von China. Jahrbücher der Deutschen Malakozoologischen Gesellschaft 14: 343–369.

[B14] Hausdorf B (2000) Biogeography of the Limacoidea sensu lato (Gastropoda: Stylommatophora): vicariance events and long-distance dispersal. Journal of Biogeography 27(2): 379–390. 10.1046/j.1365-2699.2000.00403.x

[B15] Heude PM (1882) Notes sur les mollusques terrestres de la vallée du fleuve Bleu. Mémoires Concernant l’Histoire Naturelle de l’Empire Chinois 1: 1–84. 10.5962/bhl.title.50365

[B16] Kerney MP Cameron RAD (1979) A Field Guide to the Land Snails of Britain and North-West Europe. Collins, London, 288 pp., 24 pls.

[B17] Klingenberg CP (2011) MorphoJ: an integrated software package for geometric morphometris. Molecular Ecology Resources 11: 353–357. 10.1111/j.1755-0998.2010.02924.x 21429143

[B18] Kobelt W (1898–1905) Heliceen. Part 12. Section 5. Bauer & Raspe. Nürnberg, 861–1226. [pls 229–299]

[B19] Kumar S Stecher G Tamura K (2016) MEGA7: Molecular Evolutionary Genetics Analysis Version 7.0 for Bigger Datasets. Molecular Biology and Evolution 33(7): 1870–1874. 10.1093/molbev/msw054 27004904 PMC8210823

[B20] Manganelli G Giusti F (1998) *Oxychilus mortilleti* (Pfeiffer, 1859): a redescription (Pulmonata, Zonitidae). Basteria 61: 123–143.

[B21] Möllendorff OF (1875a) Chinesische Landschnecken. Jahrbücher der Deutschen Malakozoologischen Gesellschaft 2: 118–126.

[B22] Möllendorff OF (1875b) Landschnecken der nordchinesischen Provinz Chili. Jahrbücher der Deutschen Malakozoologischen Gesellschaft 2: 214–220.

[B23] Möllendorff OF (1883) Materialien zur Fauna von China. Jahrbücher der Deutschen Malakozoologischen Gesellschaft 10: 356–383.

[B24] Neubert E (1998) Annotated checklist of the terrestrial and freshwater molluscs of the Arabian Peninsula with descriptions of new species. Fauna of Arabia 17: 333–461.

[B25] Páll-Gergely B Hunyadi A Chen Z Lyu Z (in press) A review of the genus *Coccoglypta* Pilsbry, 1895 (Gastropoda: Pulmonata: Camaenidae). Zoosystema.

[B26] Raheem DC Taylor H Ablett J Preece RC Aravind NA Naggs F (2014) A systematic revision of the land snails of the Western Ghats of India. Tropical Natural History, Supplement 4: 1–294.

[B27] Rohlf FJ (2004) tpsUtil, file utility program, version 1.26. Department of Ecology and Evolution, State University of New York at Stony Brook.

[B28] Rohlf FJ (2005) tpsDig, digitize landmarks and outlines, version 2.05. Department of Ecology and Evolution, State University of New York at Stony Brook.

[B29] Ronquist F Teslenko M Van der Mark P Ayres DL Darling A Hoehna S Larget B Liu L Suchard MA Huelsenbeck JP (2012) MrBayes 3.2: efficient Bayesian phylogenetic inference and model choice across a large model space. Systematic Biology 61(3): 539–542. 10.1093/sysbio/sys029 22357727 PMC3329765

[B30] Schileyko AA (2002) Treatise on recent terrestrial pulmonate molluscs. Part 9. Helicarionidae, Gymnarionidae, Rhysotinidae, Ariophantidae. Ruthenica, Supplement 2: 1167–1307.

[B31] Schileyko AA (2003) Treatise on recent terrestrial pulmonate molluscs. Part 10. Ariphantidae, Ostracolethidae, Ryssotidae, Milacidae, Dyakiidae, Staffordiidae, Gastrodontidae, Zonitidae, Daudebardiidae, Parmacellidae. Ruthenica, Supplement 2: 1309–1466.

[B32] Schilthuizen M Haase M Koops K Looijestijn SM Hendrikse S (2012) The ecology of shell shape difference in chirally dimorphic snails. Contributions to Zoology 81(2): 95–101. 10.1163/18759866-08102004

[B33] Sysoev A Schileyko A (2009) Land Snails and Slugs of Russia and Adjacent Countries. Pensoft Publishers, Sofia/Moscow, 312 pp., 142 pls.

[B34] Tamura K Stecher G Peterson D Filipski A Ko S (2013) MEGA6: Molecular Evolutionary Genetics Analysis version 6.0. Molecular Biology and Evolution 30: 2725–2729. 10.1093/molbev/mst197 24132122 PMC3840312

[B35] Tryon GW (1886) Manual of Conchology: Structural and Systematic with Illustrations of the Species. Second series: Pulmonata 2: 1–265, 64 pls.

[B36] Wade CM Mordan PB (2000) Evolution within the gastropod molluscs; using the ribosomal RNA gene-cluster as an indicator of phylogenetic relationships. Journal of Molluscan Studies 66: 565–570. 10.1093/mollus/66.4.565

[B37] Yen TC (1939) Die chinesischen Land-und Süsswasser-Gastropoden des Natur-Museums Senckenberg. Abhandlungen der Senckenbergischen Naturforschenden Gesellschaf 444: 1–234. [16 pls]

[B38] Zilch A (1968) Die Typen und Typoide des Natur-Museums Senckenberg, 41. Archiv für Molluskenkunde 98 (3/4): 155–212.

[B39] Zilch A (1974) Vinzenz Gredler und die Erforschung der Weichtiere Chinas durch Franziskaner aus Tirol. Archiv für Molluskenkunde 104 (4/6): 171–228. [pls 7–9]

